# Safety and efficacy of three trypanocides in confirmed field cases of trypanosomiasis in working equines in The Gambia: a prospective, randomised, non-inferiority trial

**DOI:** 10.1371/journal.pntd.0007175

**Published:** 2019-03-22

**Authors:** Alexandra G. Raftery, Saloum Jallow, Jean Rodgers, David G. M. Sutton

**Affiliations:** 1 The Weipers Centre Equine Hospital, Large Animal Clinical Science and Public Health, School of Veterinary Medicine, College of Medical, Veterinary and Life Sciences, University of Glasgow, Glasgow, United Kingdom; 2 Gambia Horse and Donkey Trust, Sambel Kunda, Central River District, The Gambia; 3 Institute of Biodiversity, Animal Health & Comparative Medicine, College of Medical, Veterinary & Life Sciences, University of Glasgow, Glasgow, United Kingdom; Institute of Tropical Medicine, BELGIUM

## Abstract

**Background:**

Globally, working equines have a continued and growing socioeconomic role in supporting the livelihoods of between 300–600 million people in low income countries which is rarely recognised at a national or international level. Infectious diseases have significant impact on welfare and productivity in this population and equine trypanosomiasis is a priority disease due to its severity and prevalence. Strategies are required to improve the prevention, diagnosis, management and treatment of trypanosomiasis in equines and more data are required on the efficacy and safety of current trypanocidal drugs.

**Methods:**

A prospective randomised, open-label non-inferiority trial was performed in The Gambia on horses and donkeys that fulfilled 2/5 clinical inclusion criteria (anaemia, poor body condition, pyrexia, history of abortion, oedema). Following randomised trypanocidal treatment (diminazene diaceturate, melarsomine dihydrochloride or isometamidium chloride), animals were observed for immediate adverse drug reactions and follow-up assessment was performed at 1 and 2 weeks. Blood samples underwent PCR analysis with specific *Trypanosoma* sp. primers. Treatment efficacy was assessed by measuring changes in clinical parameters, clinicopathological results and PCR-status post-treatment after evaluating for bias. Using PCR status as the outcome variable, non-inferiority of isometamidium treatment was determined if the upper bound limit of a 2-sided 95% CI was less than 10%.

**Results:**

There was a significant beneficial effect upon the *Trypanosoma* sp. PCR positive population following trypanocidal treatment for all groups. The findings of clinical evaluation and PCR status supported a superior treatment effect for isometamidium. Melarsomine dihydrochloride efficacy was inferior to isometamidium. There were immediate, self-limiting side effects to isometamidium in donkeys (26%). Diminazene had the longest duration of action as judged by PCR status.

**Conclusions:**

The data support the continued use of isometamidium following careful dose titration in donkeys and diminazene for trypanosomiasis in equines using the doses and routes of administration reported.

## Author summary

Equine trypanosomiasis is endemic in many areas of the world with high morbidity and mortality in affected populations. Trypanocides form an essential part of current treatment strategies but evidence regarding efficacy in equines is scarce. In order to inform disease management, the efficacy of three trypanocidal drugs was assessed in horses and donkeys that fulfilled 2/5 clinical inclusion criteria for trypanosomiasis in The Gambia. Selected equines received randomised treatment with either isometamidium, diminazene or melarsomine dihydrochloride and were observed for adverse drug reactions. Follow-up was performed at 1 and 2 weeks. Blood collected at each timepoint was analysed for *Trypanosoma* spp. using a PCR approach. Within the selected population 66% were PCR positive pre-treatment for *Trypanosoma* spp.. Trypanosome positive individuals responded favourably to each treatment, but clinical evaluation and PCR status post-treatment supported a superior effect for isometamidium. Melarsomine dihydrochloride had inferior efficacy to isometamidium. Immediate adverse side effects were only documented following isometamidium administration in donkeys (26%) and these were self-limiting. Diminazene had the longest duration of action as judged by PCR status. The data support the continued use of isometamidium and diminazene but not melarsomine dihydrochloride for trypanosomiasis in equines at the doses and routes of administration reported.

## Introduction

Working equines are an essential component of infrastructure, maintaining the health and welfare of between 300 and 600 million people globally, often within the most vulnerable communities [1]. Working equines are relied upon for transport and traction [2], contribute significantly to household income [3–5] and create opportunities for women and children [4,6]. The FAO (2014) [7] estimates the world equine population (mules, donkeys and horses) to be just over 127 million and approximately 85% in low income countries like The Gambia [8]. International acknowledgment of the positive impact of working equines upon poverty reduction, gender equality and environmental stability [9,10] has refocused attempts to tackle the myriad of issues such as infectious diseases which are known to compromise their welfare and productivity [1,9–11].

Prevalent diseases such as trypanosomiasis, piroplasmosis and African horse sickness have the greatest impact upon working equines due to their geographical distribution across lower income countries. Equine trypanosomiasis has been highlighted as a priority disease for which strategies are required to improve diagnosis, management and treatment [10]. Equine trypanosomiasis is caused by protozoan parasites of the genus *Trypanosoma* and encompasses three disease syndromes with overlapping clinical signs. These syndromes are clinically defined by the mode of transmission which also determines their geographical distribution [12]. Nagana (*Trypanosoma vivax*, *Trypanosoma congolense* and/or *Trypanosoma brucei subspecies*) is transmitted by the tsetse fly (*Glossina* spp.); Surra (*T*. *b*. *evansi*) is mechanically transmitted by biting flies, and Dourine (*T*. *b*. *equiperdum*) is venereally transmitted [12].

The Gambia is typical of countries situated within the tsetse belt with respect to the mix of equine species, routine husbandry and concurrent disease and management problems [13–18]. Equine trypanosomiasis is hyperendemic within this region [19–22] and mortality is high [17]. Trypanocidal treatment efficacy and safety in equines is currently poorly understood due to a predominant focus on livestock trypanosomiasis and available studies do not take into account species-specific effects or pharmacokinetics [23–35].

The aim of this study was to determine the field efficacy and safety of three widely used trypanocides; isometamidium, diminazene and melarsomine dihydrochloride in horses, donkeys and mules in The Gambia. The study showed that the response to trypanocidal treatment varied significantly depending not only on the trypanocidal drug used but also on the infective species of trypanosome present.

## Methods

### Recruitment of study animals

Subjects were recruited at two time points (November; 2012 and 2013) from 10 villages in the Central River District in The Gambia. At each village, recruitment was via a one day mobile veterinary clinic. All owners were invited to present their equines either for a free health check or because of pre-existing health concerns. Equines presenting were examined by an experienced equine vet (AR, DS). A detailed history was obtained via Gambia Horse and Donkey Trust staff acting as translators. Age was estimated from dentition [18], body condition (0-5/5) was scored [18,36] and body weight (kg) was estimated using a validated nomogram [36–38]. A 6 ml jugular blood sample was taken via direct venepuncture for measurement of haematocrit (HCT %) and total plasma protein (TP g/l). Excess blood was stored in EDTA, initially at 4°C. For animals subsequently included in the treatment trial, 0.2 ml of whole blood was placed upon a Whatman FTA Classic card, as a contingency for lost or damaged whole blood samples, and the remainder of the EDTA blood tube was stored at -20°C for later PCR analysis. Excess blood was discarded after measurement of HCT and TP if the animal was not included in the study.

Animals were included in the study if at least two of five clinical inclusion criteria were fulfilled: body condition score ≤1.5/5, HCT ≤24%, temperature horse >38.5°C, donkey >37.8°C, limb or ventral oedema and a history of abortion at any time prior to the study period. Animals were excluded if they had a concurrent debilitating condition, a primary presenting sign of neurological disease or had received trypanocidal treatment in the previous month. A debilitating condition was defined as a concurrent severe condition such as a long bone fracture or severe soft tissue injury likely to result in death or necessitate euthanasia during the study period. Information about previous trypanocidal treatment was acquired from the owner of the animal.

### Enrolment and treatment

Verbal informed consent was obtained from the owner for inclusion in the study. A microchip with a unique identifying number was placed in the mid third of the nuchal ligament at the time of inclusion. The animal received a randomised treatment (melarsomine dihydrochloride, diminazene, or isometamidium) using simple randomisation that was concealed from those enrolling animals onto the study. At the time of the study, all three of the trypanocides were in common use in that region for treatment of equine trypanosomiasis and there was no clinical evidence to support superiority of one trypanocidal drug compared to another, or an increased risk of administration to a particular subgroup.

After treatment the animal was observed for any immediate side effects such as type 1 hypersensitivity reactions or acute toxicity. A summary of drugs and routes of administration is detailed in Table 1. Due to differences in gross appearance and route of administration of the trypanocides the investigators could not be blinded to the treatment received but follow up examinations were blinded to prior treatment allocation.

**Table 1:** Summary of the doses and routes of administration selected for the three trypanocides.

DRUG TRADE NAME	COMPOUND	DOSE (MG/KG)	ROUTE OF ADMINISTRATION	CONCENTRATION (%)
**DIMINASAN**	Diminazene diaceturate	3.5 mg/kg	I.M. split into two aliquots one in each rump; using a 20g 1.5 inch needle.	5%
**CYMELARSAN**	Melarsomine dihydrochloride	0.25 mg/kg	I.V. 19g 1.5 inch needle.	0.5%
**SAMORIN**	Isometamidium chloride	0.5 mg/kg	I.V. 19g 1.5 inch needle	0.5%

Drugs were sourced directly from the pharmaceutical company or from a verified reputable supplier at the described dosages and administration routes.

Animals were re-examined at 1 and 2 weeks post-treatment, and history, clinical examination and blood sampling were repeated. If clinical response to treatment had been poor [two out of three of anaemia ≤ 24%, pyrexia (horse >38.5°C, donkey>37.8°C) or dull demeanour still present] the treatment was repeated with a different trypanocide (isometamidium or if used previously, diminazene) at the time of the second re-examination.

### Sample processing

Centrifugation (SpinCrit Centrifuge) of micro-haematocrit capillary tubes was used to measure the HCT. TP was measured on plasma using a hand held refractometer (Sinotech N-style) that had been pre-calibrated with sterile deionised water.

### DNA extraction

Genomic DNA was extracted from the whole blood samples using QIAamp DNA Blood Maxi Kit (QIAGEN) following manufacturer’s instructions [39] [with the substitution of 50 μl Proteinase K (20 mg/ml) for the supplied Qiagen Protease]. The purified DNA was eluted from the column in a volume of 600 μl Buffer AE. In the absence of whole blood samples, genomic DNA was extracted from the Whatman FTA cards using the QIAamp DNA Micro Kit (QIAGEN) for gDNA extraction [40]. To compensate for the uneven distribution of trypanosomes across a blood spot on an FTA card [41] 3 extractions each using 3 x 2mm punches were taken from each card to determine the infective status for each sample. For a given individual the same type of sample was used for each time point.

### PCR analysis

To confirm that the extracted DNA was of suitable quality for PCR analysis the assay was performed employing an equine specific (*Equus ferus caballus* or *Equus africanus asinus*) primer pair targeting a highly conserved equine cytochrome b sequence [42].

To detect *Trypanosoma* spp. DNA, primers [43] targeting the multicopy number satellite DNA located on the mini chromosomes, were selected due to the high sensitivity and specificity. The *Trypanosoma* sp. and subspecies of interest were selected based upon previous surveys indicating their presence in this region [19,20]. These primers, *Trypanosoma congolense* Savannah (TCS 1 and 2), *Trypanosoma brucei* sp. (TBr1 and 2) and *Trypanosoma vivax* West (TVW1 and 2) are the current gold standard diagnostic as recommended by the OIE [44] and sequence details are provided in Table 2.

**Table 2:** Sequence of primer pairs and cycling parameters employed in PCR analysis.

	CYTOB	TBR	TCS	TVW
FORWARD PRIMER	GACCTCCCAGCTCCATCAAACATCTCATCTTGATGAAA	GAATATTAAACAATGCGCAG	CGAGAACGGGCACTTTGCGA	CTGAGTGCTCCATGTGCCAC
REVERSE PRIMER	CTCAGATTCACTCGACGAGGGTAGTA	CCATTTATTAGCTTTGTTGC	GGACAAACAAATCCCGCACA	CCACCAGAACACCAACCTGA
ANNEALING TEMPERATURE	60°C	55°C	60°C	62°C
NUMBER OF CYCLES	32	35	35	30^a^/35^b^
AMPLICON SIZE	439bp	164bp	315bp	150bp

CytoB, cytochrome B; TBr, *T*. *brucei*; TCS, *T*. *congolense* Savannah; TVW, *T*. *vivax* West.

^a^Cycle number employed for analysis of DNA extracted from whole blood.

^b^Cycle number used following DNA extraction from FTA card.

PCR amplification was conducted in a total reaction volume of 25 *μ*l comprising 2.5 *μ*l template DNA, 1 x HP buffer, 0.25 mM dNTPs, 1.5mM MgCl_2_, 0.01 *μ*g/*μ*l Fw primer, 0.01 *μ*g/*μ*l Rv primer and 1.25U Taq Thermo Hotstart. Positive and negative controls were used to minimise the probability of false positive or negative results. Positive controls were stock samples of laboratory strains (*T*. *b*. *brucei* GVR35; *T*. *congolense* gifted L.M., *T*. *vivax* gifted L.M.). PCR was then performed. For all primer pairs amplification was initiated by a single cycle of 95°C for 15 min. This was followed by denaturation at 94°C for 15 s, annealing (temperature dependent on primer pair) for 30 s and extension at 94°C for 90 s. Primer specific cycling parameters used are detailed in Table 1. In each case a final extension at 72°C for 10 min was incorporated.

Following amplification, for each sample 10 μl of PCR product was loaded on to a 2% agarose gel (SeaKem) containing 1 μg/ml ethidium bromide. 5 μl of 100 bp ladder (Invitrogen) was used to assess the PCR product size. The electrophoresis was performed for 45 min at 150V in 1 x TBE buffer prior to examination and imaging in a UV transilluminator.

### PCR analysis for concurrent haemoparasite infection

The samples from week one (initial examination) were also screened by nested PCR with a modified Babesia/Theileria 18s catch-all primer set [45–47]. Reaction conditions were an initial denaturation at 94°C for 5 min, followed by 30 cycles of 94°C for 45 s, annealing at 67°C (external primers) or 57°C (internal primers) for 60 s, elongation at 72°C for 60 s, with a final extension at 72°C for 5 min. A 1:10 dilution of the primary reaction product was used as a template for the secondary reaction.

A subsection of the week 1 samples (n = 60) was screened by nested PCR for *Anaplasma* sp. using a catch all primer set [48]. Reaction conditions were an initial denaturation at 94°C for 5 min, followed by 30 cycles of 94°C for 30 s, annealing at 65°C (external primers) or 64°C (internal primers) for 30 s, elongation at 72°C for 60 s, with a final extension at 72°C for 5 min. A 1:10 dilution of the primary reaction product was used as a template for the secondary reaction.

### Interpretation of clinical and clinicopathological data

Species specific reference ranges were identified for body temperature (donkey 36.2–37.8; horse 37.7–38.5°C), heart rate (donkey 40–53; horse 24–40 beats per min) and respiratory rate (donkey 16–20; horse 6–16 breaths per min) [49,50]

Species specific reference values for HCT for horses (31–43%) [51] and donkeys (27–42%) [52] from UK equine populations were selected since there are no endemic infectious diseases resulting in anaemia.

### Statistical analysis

Sample size calculations for non-inferiority analysis were based upon the most recent published prevalence data for *Trypanosoma* spp. available for the region [19] and previous published efficacy for the two drugs anticipated to have the least disparity based upon available literature (diminazene, isometamidium).

Assuming a treatment success of 100% (isometamidium)[23] and 82% (diminazene) [17,18,28] with a two- sided significance (α) of 0.05 and a power of 0.8 ((P = 1-ß); ß = 0.2) a total of 14 animals positive for each *Trypanosoma* sp. was required [53,54]. To ensure representation of all three pathogenic *Trypanosoma* spp. (*i*.*e*. to ensure 14 positive of each *Trypanosoma* sp.) the total required group size was increased to 78 (assuming 18% positive for *T*. *brucei* sp. in population presenting for treatment [19]). Assuming 30% may be lost to follow up a minimum of 100 animals was required to be recruited on week 1.

Rstudio was used for statistical analysis. Normality testing was performed with the Shapiro-Wilk test. Median and inter-quartile range were reported for numerical non-parametric data, mean and standard deviation for parametric data. The Wilcoxon signed rank test was used for comparison of paired non-parametric data and the Wilcoxon rank sum test for independent non-parametric data. The Kruskal-Wallis test was used to compare more than two groups of non-parametric data. Chi-squared test was used to compare two proportions of categorical data or the Fischer’s exact test where group size was small (difference less than 5). For comparison of paired proportions, the McNemar test was used. The level of significance was set as p≤0.05.

Non-inferiority analysis was used to determine treatment efficacy as outlined by the FDA for clinical trials [55] with PCR status as the outcome variable. As the most frequently used trypanocide within The Gambia, isometamidium was selected as the control drug. M1 (the difference between the control drug and a placebo controlled study) was estimated at 60% from a previous study which had assessed the effect of using isometamidium as prophylaxis compared to no treatment [24]. A value of 10% was selected for M2 (the accepted difference between the control drug and a test drug) based upon clinical experience and previously published treatment trials [56]. For each *Trypanosoma* sp. (*T*. *vivax*, *T*. *congolense*, *T*. *brucei* sp.), at each time point (week 2, week 3), the difference (%) in achieving a positive outcome between the control drug and the test drug (point estimate) and 95% confidence intervals were calculated. The analyses were repeated on a whole animal basis defining treatment success as PCR negative for all *Trypanosoma* spp. following treatment. Each analysis was visualised on a forest plot.

### Ethics statement

Ethical approval for this study was granted by the University of Glasgow School of Veterinary Medicine ethics committee (reference 21a/13), The Donkey Sanctuary ethics board and the Gambian Ministry of Agriculture. The study abided by the Veterinary Surgeons Act 1966 (this was the criteria used by the committee), procedures were performed by a veterinary surgeon and the procedures were of direct benefit to the animal (diagnostic blood samples) and so the study did not come under Animals (Scientific Procedures) Act 1986. Verbal consent was obtained from the owner for inclusion in this study and documented on the dedicated form.

## Results

**Fig 1:**
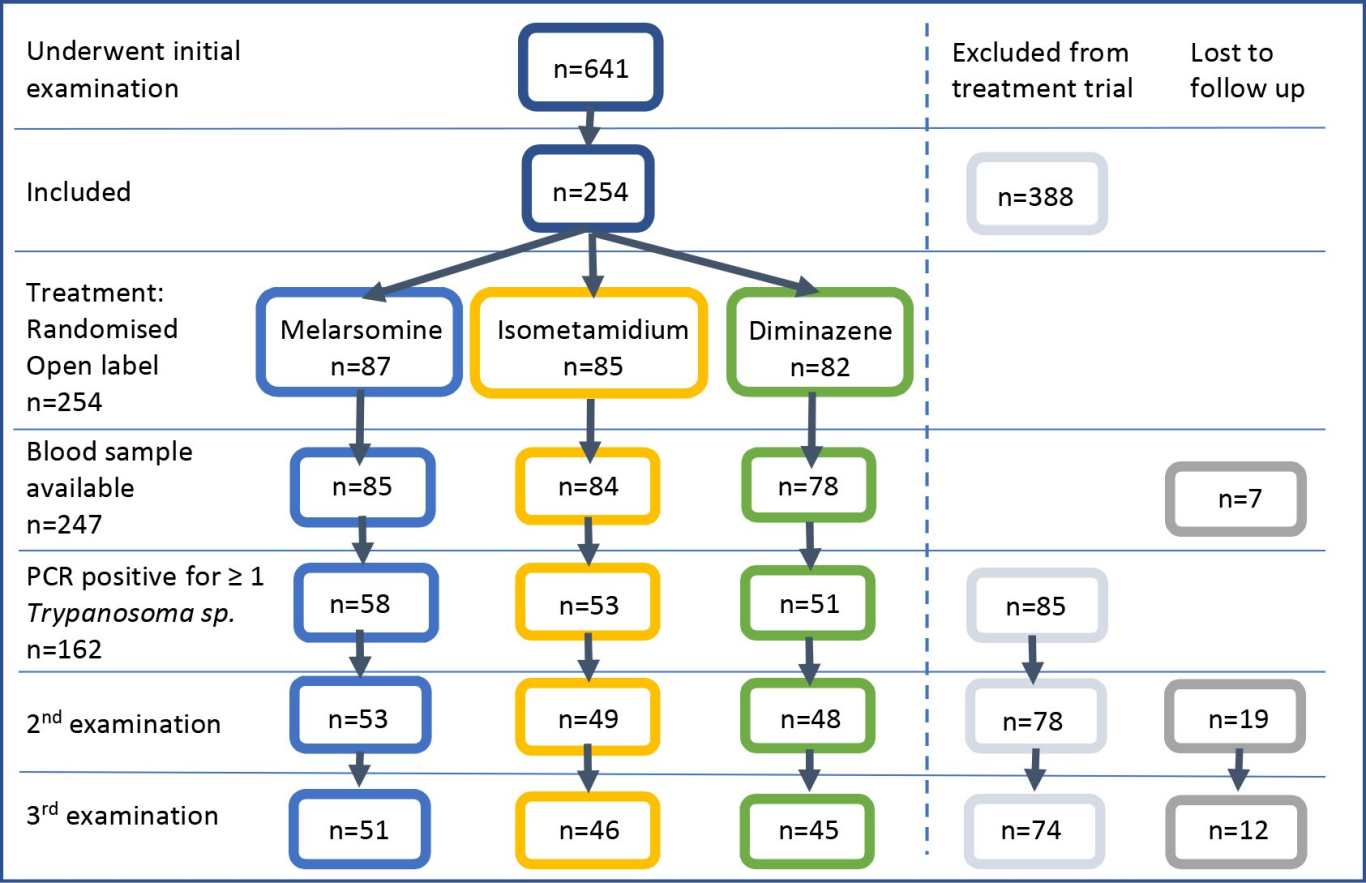
Flow chart illustrating recruitment of equines to the treatment study. Of the 254 animals which fulfilled clinical criteria for inclusion in the study, 162 subsequently proved PCR positive for at least one *Trypanosoma* sp. and were included in the treatment trial (n = 162). Animals that were PCR negative for all *Trypanosoma* sp. at the first examination were excluded from the treatment trial (n = 85). Adverse drug reactions were documented for the whole treated population (n = 254). Animals which fulfilled the clinical criteria for which PCR status was known (n = 247) were used to document the number of new or recrudescence of existing infections within treatment groups. Colours correspond to treatment group (blue = melarsomine, yellow = isometamidium, green = diminazene), exclusion (grey) or loss to follow up (dark grey) of animals.

Animals from the whole treated population (n = 254) that were positive on PCR for at least one *Trypanosoma* sp. (n = 162) were used to assess the relative treatment efficacy of the three selected trypanocides (Fig 1).

### Baseline parameters

Baseline history, signalement, subjective and objective clinical examination findings and clinicopathological results for the treatment trial population (n = 162) were comparable between treatment groups (p>0.05) and are summarised in supplementary data (S1 Table). A quiet demeanour was common in individuals with *Trypanosoma* sp. infection (84/162; 52%); smaller numbers were assessed as dull (34/162; 21%) or bright (32/162; 20%). Oedema was an uncommon finding (6/162; 4%) in infected individuals but tachycardia was extremely common (124/162; 77%). Within the female population previous abortion, occurring at any time prior to presentation, was commonly reported (21/94; 22%), with no difference between treatment groups. This was representative of the sampled population (60/340; 18% of female equines) (p = 0.3). A history or clinical evidence of diarrhoea was found in 7/162 (4%) of animals which was representative of the sampled population (17/639; 3%) (p = 0.3). All animals were positive for at least one *Trypanosoma* sp. [*T*. *congolense* (110/162; 68%)*; T*. *vivax* (80/162; 49%) *or T*. *brucei* sp. (38/162; 23%)] and co-infection was common (52/162; 32%). More than half of the animals (90/162; 56%) were also positive for piroplasmosis, the proportion did not differ between treatment groups (p>0.05). None of the tested animals were positive for anaplasmosis (0/60; 0%).

### Treatment effect on clinical parameters

#### Subjective parameters

**Fig 2:**
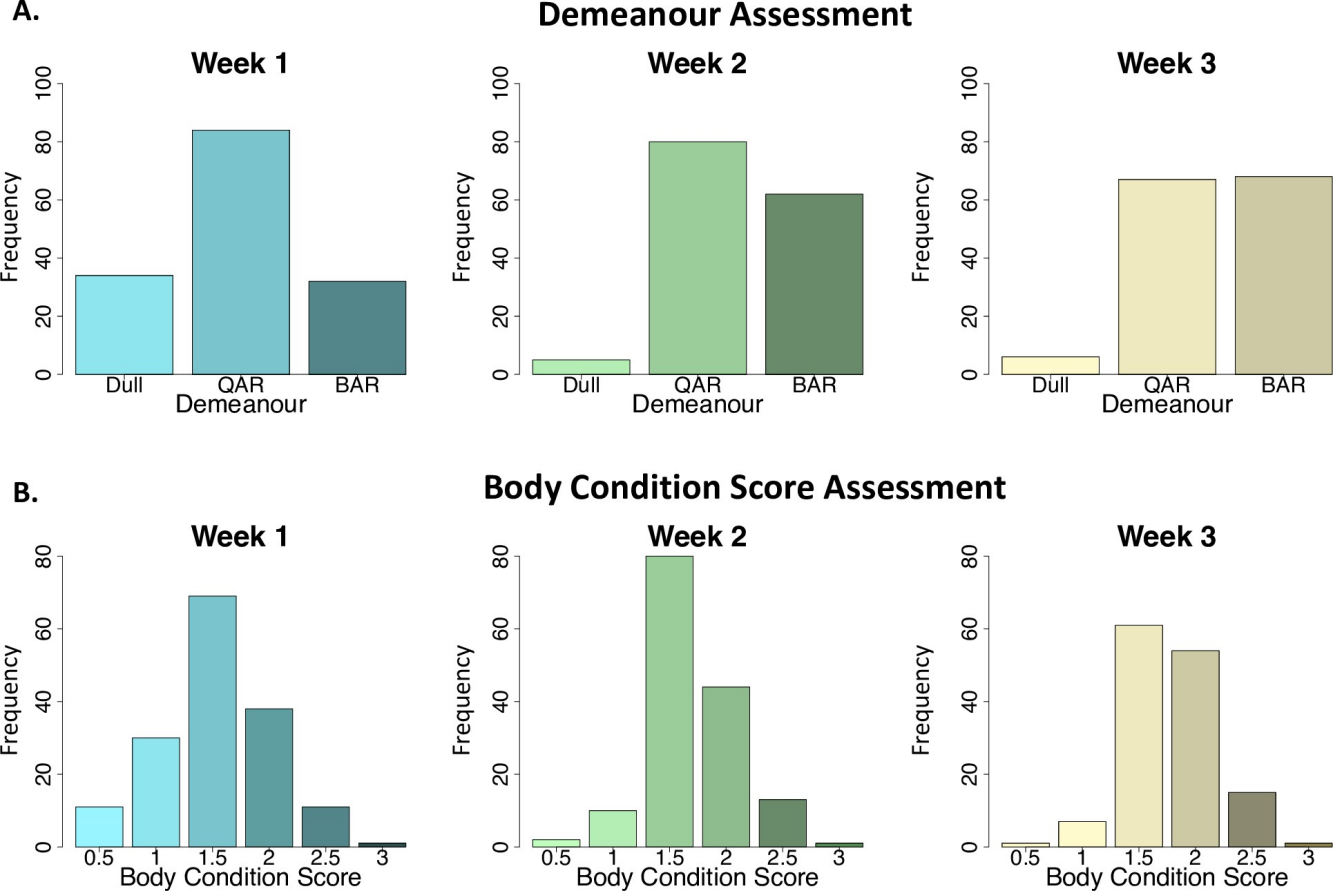
Bar plots illustrating categorisation of subjective parameters for the treatment trial population (n = 162). Demeanour (A) and body condition score (B) were evaluated during the initial examination (week 1) and follow up post-treatment evaluations (weeks 2 and 3). Number of individuals (frequency) is plotted on the y-axis in each case. Bar plots represent the whole treatment trial population at each time point (week 1, week 2 and week 3). Demeanour (A) was assessed as BAR (bright, alert and responsive), QAR (quiet, alert and responsive) or dull. Assessed demeanour improved following treatment (week 1 to week 2) (p<0.001). Body condition (B) score (0-5/5) improved following trypanocidal treatment (p = 0.003).

In the treatment trial population (n = 162) there was a general improvement in the assessed demeanour following treatment (Fig 2A) with a greater number of bright animals (68/141; 48%) and fewer dull animals (6/141; 4%) in week 3 compared to week 1 (p<0.001). A significantly greater effect on demeanour was noted for isometamidium and diminazene at week 2 compared to melarsomine dihydrochloride (p = 0.007), but this difference was no longer evident by week 3 (p = 0.26) (S4 Table). There was also a general improvement in the assessed body condition score following treatment (p = 0.003) (Fig 2B). The median body condition score prior to treatment was 1.5/5 (IQR 1–2) with an increase to 2/5 (1.5–2) by week 3 and this did not vary significantly between treatment groups (p = 0.47).

#### Objective clinical parameters

**Fig 3:**
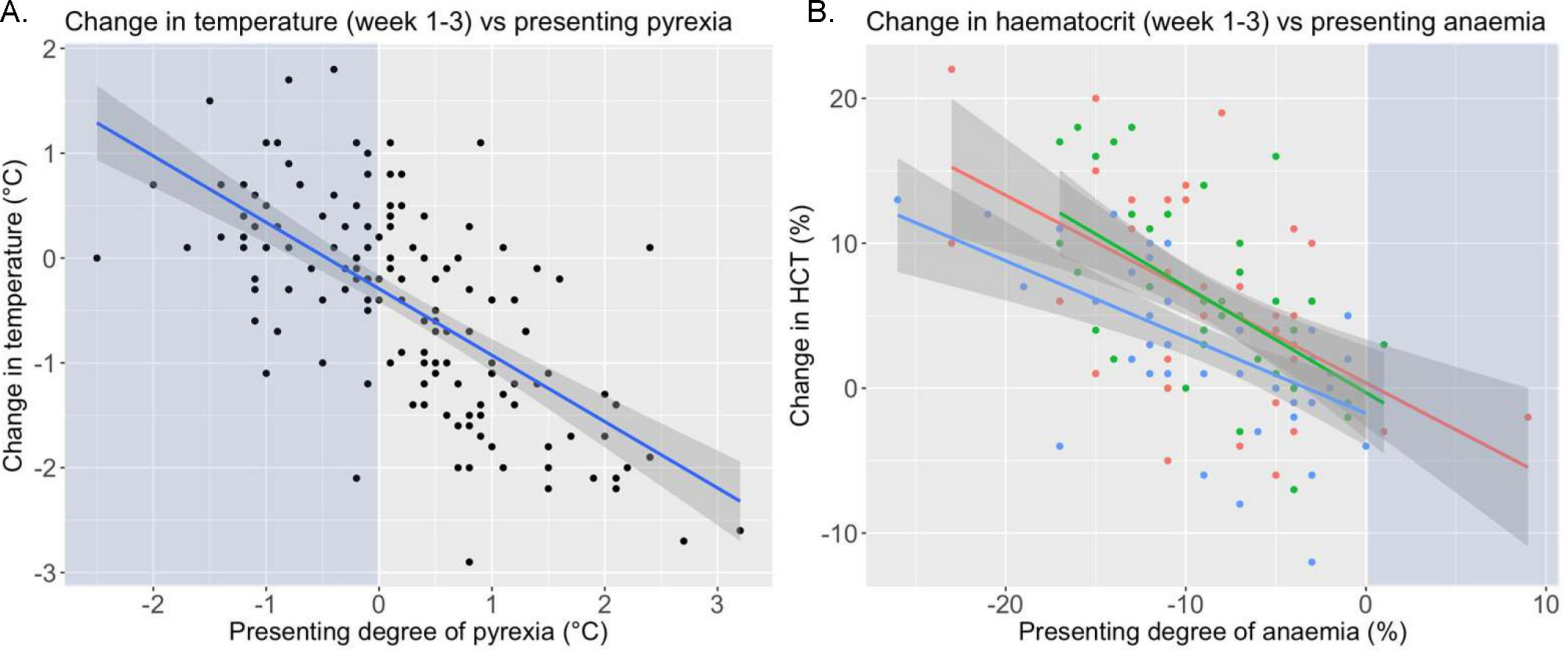
Scatterplots illustrating the change in clinical parameters from week 1 to 3. A. Scatterplot showing individual changes in rectal temperature for whole treatment trial population (n = 162) following treatment. The regression line is marked in blue with 95% confidence intervals in dark grey. Light blue background shading illustrates animals presenting with parameters within reference range. B. Scatterplot showing individual changes in HCT, with colour coding to illustrate trypanocidal treatment group (melarsomine dihydrochloride = blue, diminazene = red and isometamidium = green) for whole treatment trial population (n = 162) following treatment. The regression line colour represents the trypanocide with 95% confidence intervals shown in dark grey. Presenting degree of anaemia (%) calculated by the difference between the animal’s HCT and the lower end of species appropriate reference values (donkey 27%; horse 31%).

A significant decrease (Fig 3) in rectal temperature (°C) over the treatment period was apparent in all groups (week 1 to week 3; p<0.001) but there were no differences between treatment groups. There was no significant change in heart rate or respiratory rate following treatment and values above reference range remained common post-treatment (S2 Table).

### Treatment effect on clinicopathological parameters

There was a significant (p<0.001) increase in haematocrit over the treatment period from pre- (week 1) to post-treatment (week 2 and week 3) for the whole treatment group. The median HCT prior to treatment was 20% (16–23), 23% (20–26) at week 2 and 25% (22–28) at week 3. Treatment with melarsomine dihydrochloride resulted in a smaller increase in HCT (3% (0–6)) (p = 0.025) than isometamidium (6% (2–10)) or diminazene (5% (1–10)) (Fig 3; Table 3).

**Table 3:** Clinicopathological parameters.

		WEEK 1			WEEK 2			WEEK 3		
PARAMETER		Cy n = 58	Dim n = 51	Iso n = 53	Cy n = 54	Dim n = 48	Iso n = 49	Cy n = 50	Dim n = 44	Iso n = 45
**HAEMATOCRIT (%)**	**Horse**	20 (19–24)	20 (13–24)	20 (15–23)	22 (20–23)	24 (19–27)	24 (21–27)	22 (18–23)	22 (20–28)	28 (24–28)
** **	**≤30%**	20/21 (95%)	16/16 (100%)	18/18 (100%)	21/21 (100%)	14/15 (93%)	16/17 (94%)	18/18 (100%)	10/11 (91%)	13/15 (87%)
** **	**Donkey**	20 (16–24)	20 (16–23)	20 (16–23)	22 (20–26)	23 (21–27)	23 (21–26)	23 (21–25)	26 (23–28)	26 (22–28)
** **	**≤26%**	37/37 (100%)	32/35 (91%)	34/35 (97%)	25/33 (76%)	24/33 (73%)	25/32 (78%)	27/ 32 (84%)	19/33 (58%)	18/30 (60%)
**TOTAL** **PROTEIN (G/L)**	**Horse**	80 (70–90)	77 (70–87)	88 (76–92)	90 (82–100)	84 (80–93)	96 (88–105)	96 (81–103)	81 (78–93)	100 (93–104)
** **	**Donkey**	82 (75–90)	82 (75–90)	80 (75–87)	98 (86–105)	96 (90–105)	98 (90–105)	100 (92–106)	96 (87–106)	95 (90–106)

Haematocrit (%) and total plasma protein (g/l) for the whole treatment population (n = 162) subdivided by trypanocidal drug group (melarsomine dihydrochloride (Cy), diminazene (Dim), isometamidium (Iso) are presented by species (horse and donkey) and time point (week 1, 2 and 3). Parameters are presented as median and IQR and categorised as within or outside the reference range (proportion and percentage).

Total plasma protein (TP g/l) increased significantly (p<0.001) from a median of 82 g/l (74–90) to 95 g/l (87–105) by week 2, remaining elevated at week 3 (96 g/l (87–105) (Table 3). There was no significant difference in response between treatment groups.

### Treatment effect on *Trypanosoma* sp. PCR status

#### Demonstration of treatment effect

**Fig 4:**
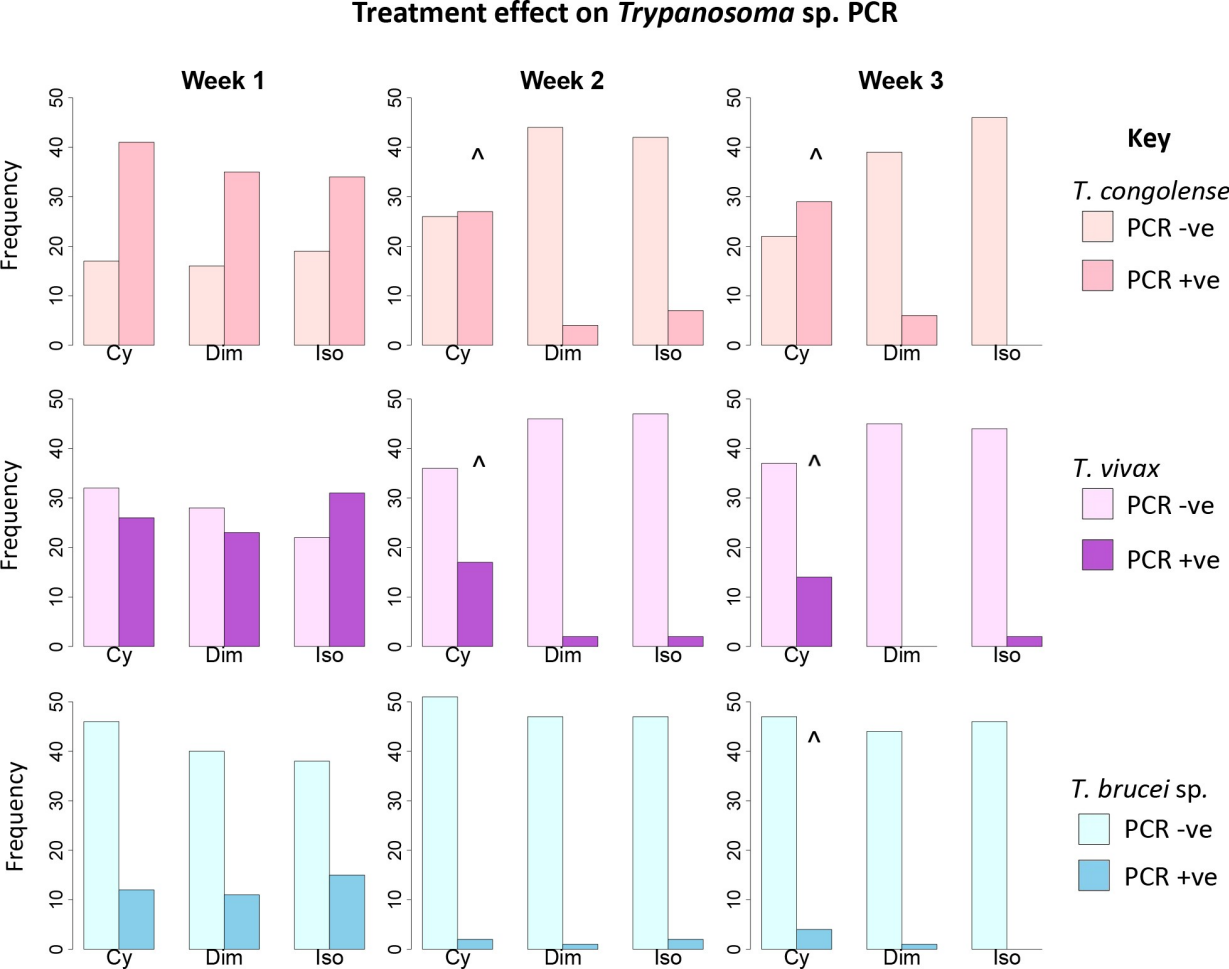
Bar chart lattice illustrating *Trypanosoma* spp. PCR status at each time point for each drug. Treatment trial group (n = 162) are subdivided by trypanocidal drug (Cy = melarsomine dihydrochloride, Dim = diminazene, Iso = isometamidium), time period (week 1, 2 or 3) and infective *Trypanosoma* spp. (*T*. *brucei* sp., *T*. *congolense* or *T*. *vivax*). ^ Denotes no significant change (p>0.05) in PCR status from week 1. Isometamidium demonstrated a comparable effect (94–100% reduction in PCR positive status) to that reported in previous studies [24,25]. This validates the use of isometamidium for use as the control drug for non-inferiority analysis to compare the outcome to trypanocidal treatment based on PCR status.

The selected trypanocidal agents caused a significant decrease in the proportion of PCR positive animals for *Trypanosoma congolense*, *T*. *vivax* and *T*. *brucei* sp. in the whole treatment trial population (p<0.001) (S3 Table, Fig 4). The reduction in PCR positive animals was not statistically significant (p>0.05) at week 2 or 3 for *T*. *congolense* and *T*. *vivax* or week 3 for *T*. *brucei* sp. in the melarsomine dihydrochloride group.

### Non-inferiority analysis of comparative trypanocidal efficacy

#### Comparison of diminazene to isometamidium

**Fig 5:**
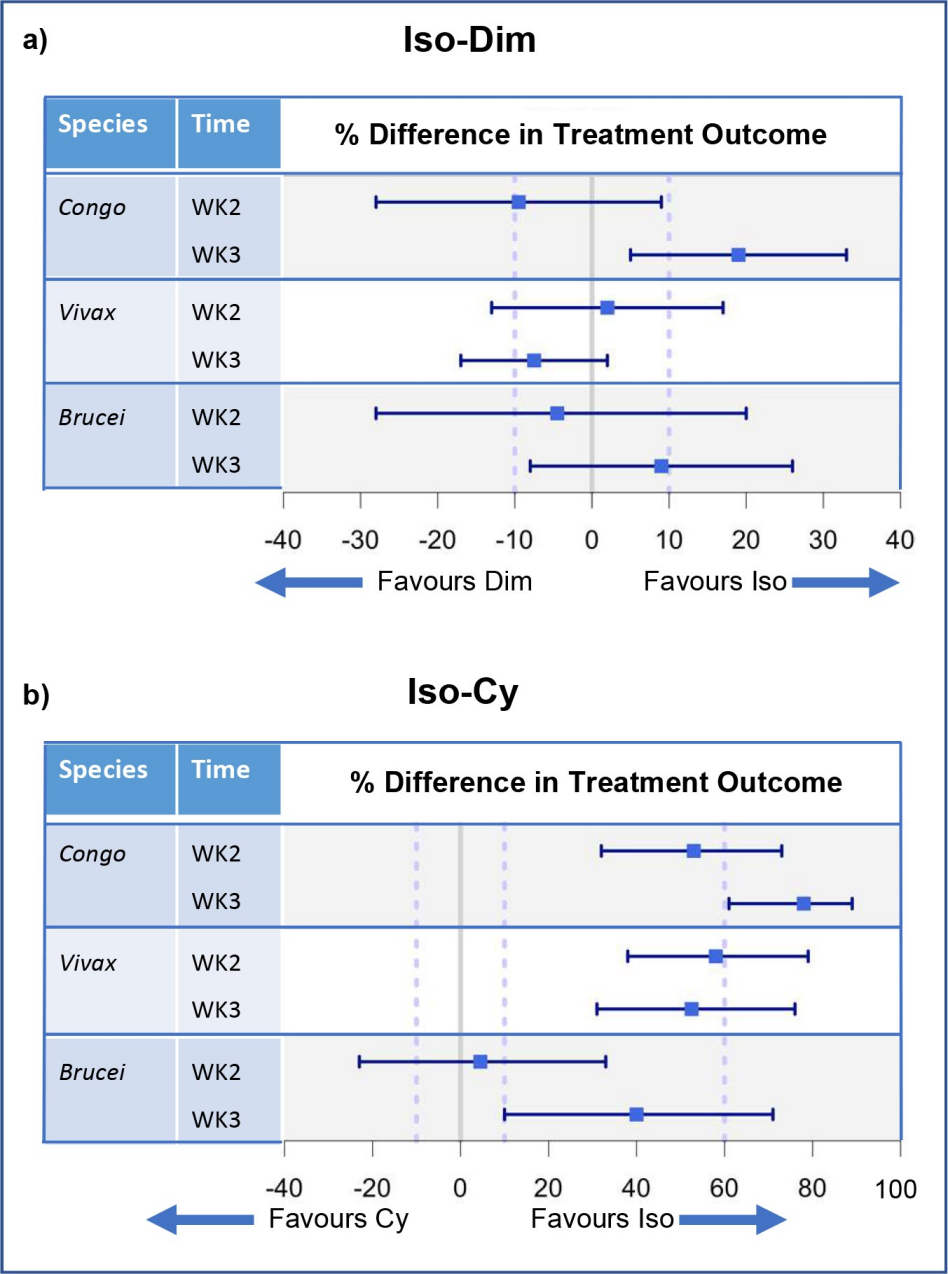
Forest plots illustrating non-inferiority analysis of the outcome of trypanocidal treatment by *Trypanosoma* spp.. *Trypanosoma* spp. PCR status was used as the outcome variable. The control drug (Iso = isometamidium) was compared to test drugs (Dim = diminazene (a) and Cy = melarsomine dihydrochloride (b)) for each infective species of *Trypanosoma* spp. at each time point (week 2 and 3). Positive treatment outcome was defined as a negative PCR result. The percentage difference in treatment outcome is presented as a point estimate (blue square) and two sided 95% confidence intervals (blue bars). Results are divided by *Trypanosoma* spp. (*Brucei* = *T*. *brucei* sp.*; Congo = T*. *congolense; Vivax = T*. *vivax*) and time point (week 2 and week 3). The 10% line represents M2, the accepted difference between drugs to demonstrate non-inferiority. The 60% line represents M1, the accepted difference between the control drug and the test drug to demonstrate placebo effect. a) Isometamidium (control drug) is compared to diminazene (test drug) b) Isometamidium (control drug) is compared to melarsomine dihydrochloride (test drug).

At week 2 diminazene was non-inferior to isometamidium for treatment of *T*. *congolense* infections. By week 3 diminazene was inferior to isometamidium as evidenced by PCR status. For *T*. *vivax* at week 2, non-inferiority of diminazene to isometamidium was not demonstrated. By week 3 diminazene treatment was non-inferior to isometamidium. For *T*. *brucei* sp. at week 2, the wide confidence intervals led to inconclusive results with respect to inferiority. At week 3 non-inferiority of diminazene was not demonstrated but could not be excluded (lower 95% CI < 0%) (Fig 5A; S5 Table).

#### Comparing melarsomine dihydrochloride to isometamidium

For *T*. *congolense* at week 2, isometamidium was superior to melarsomine dihydrochloride. The results did not support a difference between melarsomine dihydrochloride and placebo administration (M1 value). At week 3 isometamidium was superior to melarsomine dihydrochloride and the effect of melarsomine dihydrochloride was equivalent to less than placebo administration (M1 value). For *T*. *vivax* at week 2 and 3, isometamidium was superior to melarsomine dihydrochloride, and the results could not support a difference between melarsomine dihydrochloride and placebo administration. For *T*. *brucei* sp. at week 2 non-inferiority of melarsomine dihydrochloride was not demonstrated but could still be plausible (lower 95% CI < 0%). At week 3 isometamidium was superior to melarsomine dihydrochloride and a comparable efficacy of melarsomine dihydrochloride to placebo could not be excluded (Fig 5B; S6 Table).

#### Whole animal PCR status

**Fig 6:**
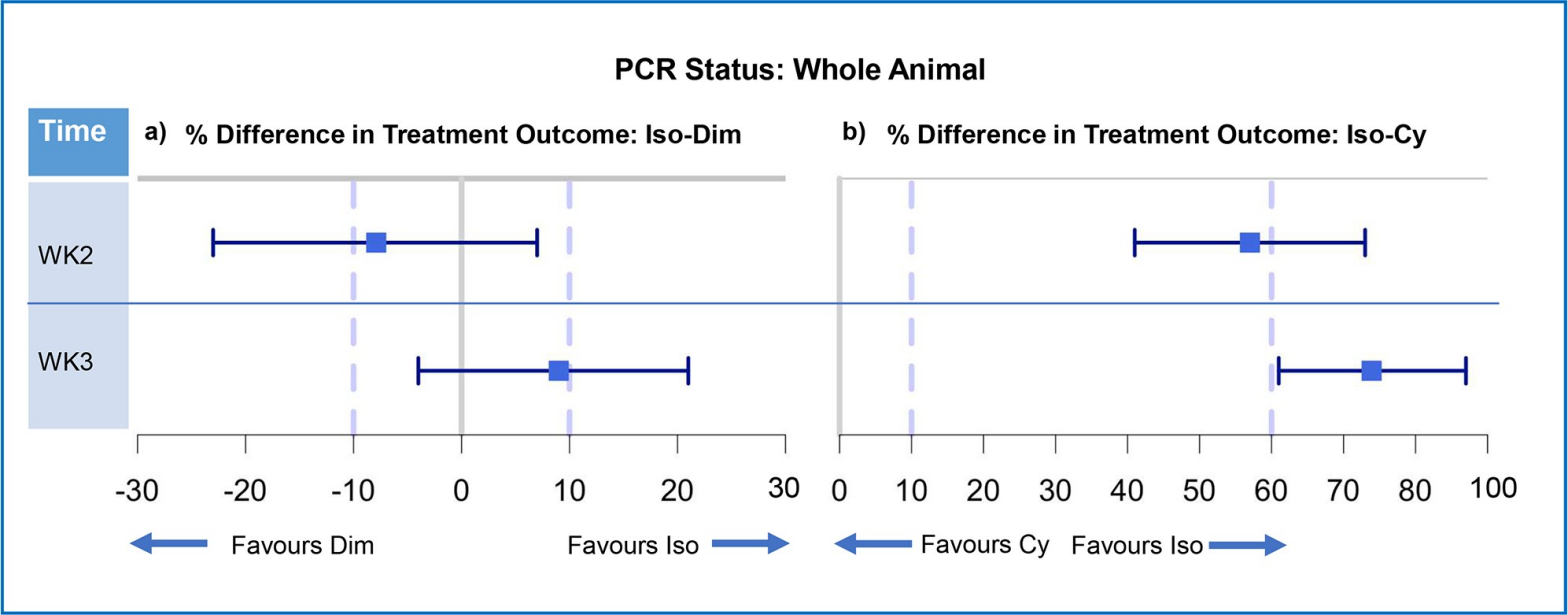
Forest plots illustrating non-inferiority analysis of the outcome of trypanocidal treatment by whole animal. Forest plots illustrating non-inferiority analysis of control drug and test drug for whole animal (n = 162). a) Control (isometamidium) vs test (diminazene) b) Control (isometamidium) vs test (melarsomine dihydrochloride). Positive treatment outcome was defined as a negative PCR result for all *Trypanosoma* spp. The percentage difference in treatment outcome was presented as a point estimate (blue square) and two-sided 95% confidence intervals (blue bars). Results are presented as whole animal *Trypanosoma* spp. status by PCR and are divided by time point (week 2 and week 3). The 10% line represents M2, the accepted difference between drugs to demonstrate non-inferiority. The 60% line represents M1, the accepted difference between the drugs to demonstrate effect comparable to placebo.

As mixed trypanosome infections were common, whole animal outcome was measured via PCR status. At week 2 diminazene demonstrated non-inferiority to isometamidium. By week 3 the point estimate favoured isometamidium and non-inferiority of diminazene was not demonstrated (Fig 6A). At week 2 isometamidium was superior to melarsomine dihydrochloride and the results could not support a difference between melarsomine dihydrochloride and placebo administration. At week 3 isometamidium was superior to melarsomine dihydrochloride and melarsomine dihydrochloride had an effect comparable to placebo (Fig 6A; S7 Table).

### New and recrudescing infection rate

**Fig 7:**
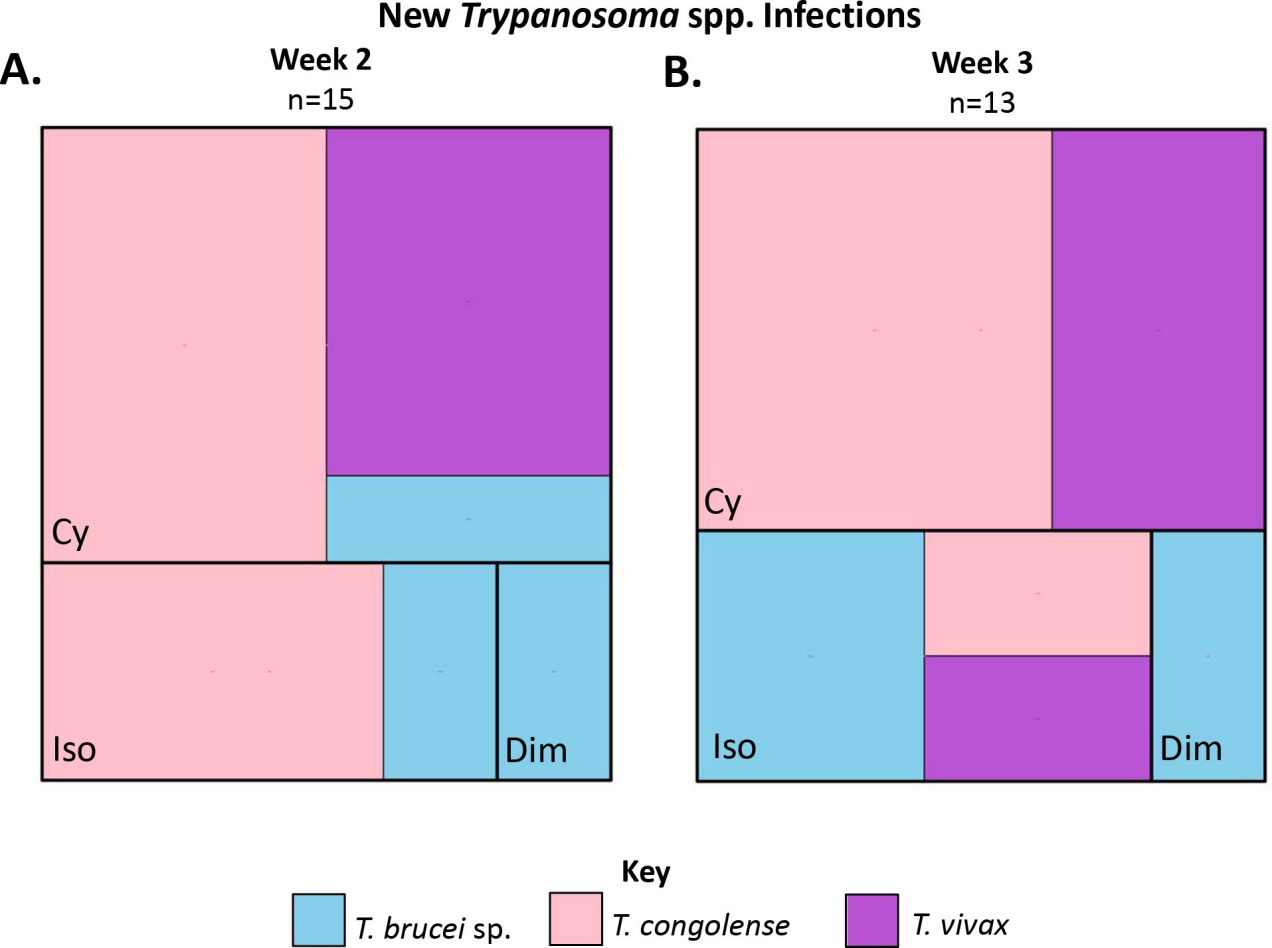
Tree maps illustrating the number of new PCR positive animals during the study period within the whole study population (n = 247). The new positives are subdivided by trypanocidal treatment (Cy = melarsomine dihydrochloride, Dim = diminazene, Iso = isometamidium) and *Trypanosoma sp* (*T*. *congolense* = pink, *T*. *brucei* sp. = pale blue, *T*. *vivax* = purple). A. Tree map illustrating new PCR positives during week 2 (n = 15). B. Tree map illustrating new PCR positives during week 3 (n = 13).

The whole treated population for which PCR results were available (n = 247) was evaluated (Fig 1). For the purpose of this analysis, new or recrudescing infections were defined as a positive PCR result at week 2 or 3 in an individual animal that was PCR negative on week 1 for that *Trypanosoma* sp. A total of 28 new or recrudescing infections occurred, the greatest number of new PCR positives occurred in the melarsomine dihydrochloride treated animals (10/87; 12%), and the fewest number of new PCR positives in the diminazene treated animals (1/85; 1%) (Fig 7).

### Adverse effects

The whole treated population (n = 254) was used to document adverse reactions to the trypanocides.

#### Immediate effects

**Fig 8:**
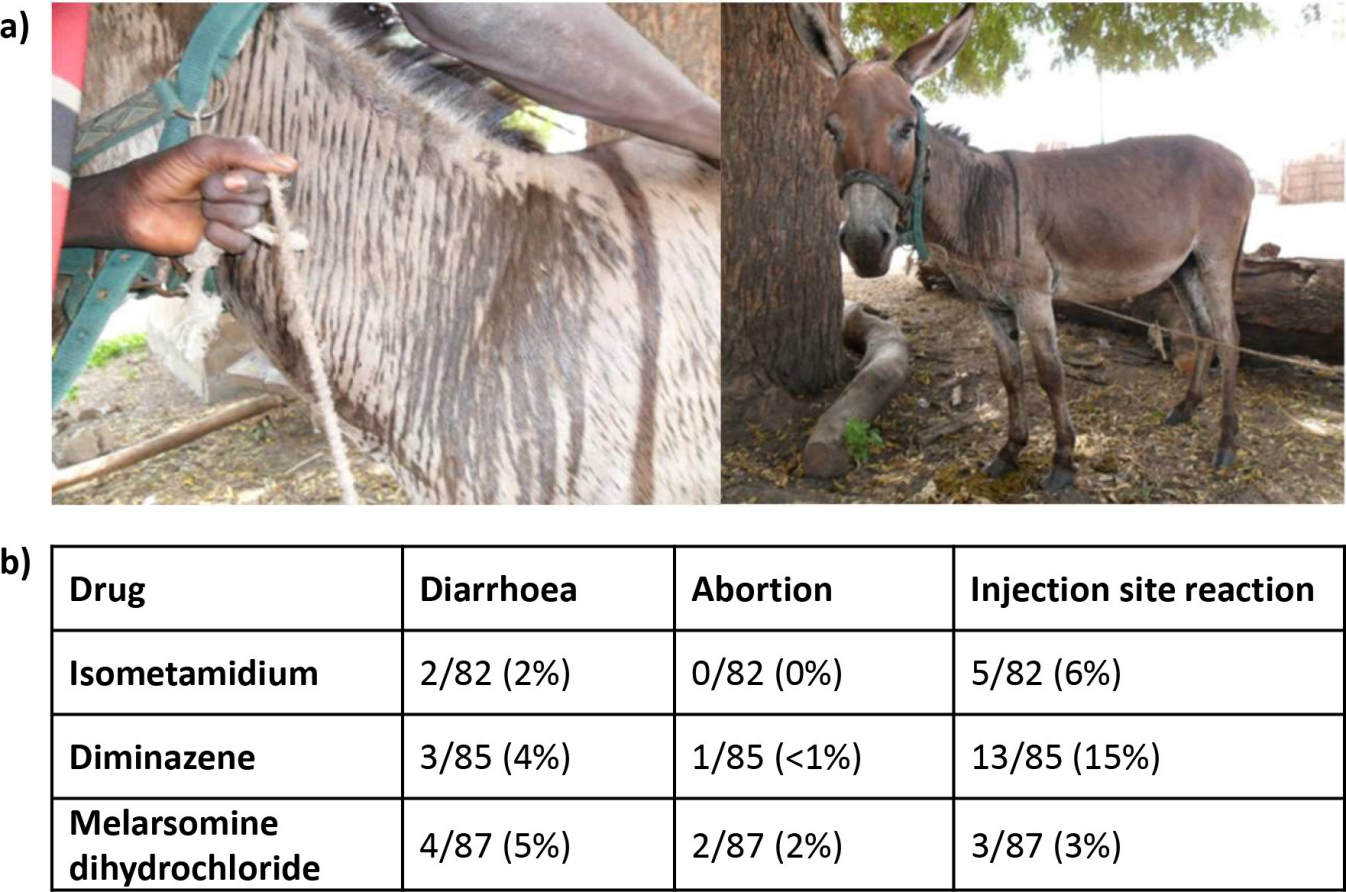
Acute and delayed adverse drug reactions occurring after trypanocidal administration in working equines. a) Acute drug reactions were only seen in donkeys. Photographs illustrate characteristic linear sweating pattern and 'skin wrinkling' observed in immediate reaction to isometamidium documented in 12/47 (26%) of treated donkeys. b) Delayed adverse reactions were seen in donkeys and horses. These are summarised in the table subdivided by treatment group.

There were no immediate adverse effects noted in horses. Immediate adverse effects were only noted in donkeys in the isometamidium treatment group (12/47 donkeys; 26%) (Fig 8A). The reactions ranged from mild to severe and occurred almost immediately following injection of the drug. No additional treatment was required as the parameters normalised over 45–60 minutes.

In the majority of these donkeys a pain response was observed (n = 9) immediately following injection. This was indicated by the animal scratching at its neck, snorting, tongue playing, tail swishing or displaying transient colic signs including rolling. These signs were transient and resolved within 5–10 minutes. Clinical signs of shock developed after treatment in 4 animals, with one severe reaction resulting in cold extremities, pale mucous membranes, profound tachycardia (84-130bpm), and increased respiratory effort and rate (100 breaths per minute). These signs gradually resolved and lasted for up to 1 hour. A characteristic skin reaction with generalised sweating and a linear sweating pattern over the neck and face associated with 'skin wrinkling' was observed in 5 animals (Fig 8A). One animal passed faeces shortly following injection and also displayed signs of the skin reaction.

#### Delayed effects

Information collected from the owners regarding any concerns following treatment after treatment together with abnormalities detected on clinical examination at weeks 2 and 3 are summarised in Fig 8B. Diarrhoea (n = 9) was self-limiting without evidence of hypovolaemia or systemic inflammatory response syndrome. Injection site reactions were mild and included perivascular reaction and/or haematoma (isometamidium and melarsomine dihydrochloride) and palpable intra-muscular swelling (diminazene). None were painful on palpation and all had resolved at the end of the study period. Abortion was owner reported and information on stage of gestation was limited.

## Discussion

This study is the first to provide a thorough, multi-faceted and comparative evaluation of the efficacy and safety of trypanocidal treatment in equines with confirmed *Trypanosoma* sp. (*T*. *vivax*, *T*. *congolense* and/or *T*. *brucei* sp.) infections, thus providing important information for development of control programs and a robust model for future field trials of trypanocides.

A positive clinical effect was documented in response to all the trypanocidal treatments. The observed positive response in demeanour and body condition score was similar between treatment groups supporting a reducing parasite burden in all groups. Weight loss in trypanosomiasis is due to a combination of reduced feed intake and impaired efficiency of feed conversion [57]. Although two weeks is a short time frame for significant body mass increase to occur [58], the documented improvement in body condition is consistent with an improved appetite, increased feed intake and intestinal fill. The median body condition score (2/5) remained poor at the final evaluation reflecting the magnitude of the disease burden and multi-morbidities such as piroplasmosis, lameness [11] and poor nutrition facing this population. A subdued demeanour has been previously reported as an indicator of acute disease onset in equines [24] secondary to pyrexia, anaemia and presence of an inflammatory response. Following trypanocidal drug treatment a large proportion of the population remained 'quiet' and did not warrant assessment as 'bright'; this may reflect incomplete resolution of disease and the potential presence of co-morbidities such as piroplasmosis.

Fever has been identified as a reliable indicator for the onset of acute *Trypanosoma* spp. infection [24] but in chronic disease this may be a less consistent finding [24]. In the present study there was a significant decrease in rectal temperature, most marked in animals originally febrile, following chemotherapy. Fever remained common (33%; 47/139) in this population post-treatment but whether this represents ongoing disease, a persistent inflammatory response due to treatment, the presence of a co-infection, the high ambient temperature or a combination of factors requires further investigation. With improved demeanour some of the animals became more challenging to handle which could have contributed to persistent tachypnoea (61%; 86/140) and tachycardia (74%; 105/142) two weeks after treatment.

Isometamidium and diminazene were the most effective of the three trypanocides in the amelioration of anaemia, but melarsomine dihydrochloride also had a positive effect. Anaemia is a major contributor to morbidity in trypanosomiasis; control of anaemia has been suggested to be more important than control of parasitaemia [59] in treating the disease phenotype. Physiological haematocrit increase is limited by the rate of erythrocyte regeneration; estimated in horses at 0.672% per day [60] which is equivalent to an increase of 9.4% over this 14 day study period. This is comparable to the maximal increase in HCT measured in the isometamidium treated horses investigated in this study. For isometamidium, the continued improvement over the two weeks after treatment supports a sustained effect in resolving or substantially reducing the mechanisms of anaemia, seen to a lesser extent for diminazene and isometamidium treated donkeys. The short duration of action of melarsomine dihydrochloride reported in other species [61] is consistent with the small increase in HCT detected at week 2 post-treatment with no further increase noted at week 3 in this treatment group. For all treatment groups it is also important to consider that the presence of concurrent diseases including piroplasmosis could contribute to the residual anaemia.

Trypanocidal treatment generates a marked inflammatory response. Total plasma protein increased in all treatment groups markedly by the first week of follow up (week 2). Differentials for hyperproteinaemia include dehydration (pan hyperproteinaemia) or hyperglobulinaemia. Clinical evaluation did not identify widespread evidence of severe dehydration however the substantial buffy coats noted following centrifugation of the blood would support a marked post-treatment inflammatory response and exacerbation of the pre-existing hyperglobulinaemia [62–64]. Since immune evasion through antigenic variation is key to parasite survival [65], treatment with a trypanocide would be expected to generate an inflammatory response as these mechanisms are then impaired. However the duration and impact of this resulting inflammatory response are unknown.

The *Trypanosoma* spp. infection status (determined by PCR) provided the greatest differentiating factor between the trypanocides in this study. The efficacy of melarsomine dihydrochloride for treatment of *T*. *congolense* in this population was comparable to placebo and was inferior to isometamidium for treatment of *T*. *brucei* sp. and *T*. *vivax*. Melarsomine dihydrochloride was also of limited efficacy in preventing new infections following treatment. Given the high challenge in the area and apparent short duration of activity a repeated dosing regimen may be more appropriate when using this drug. The results of this study indicate that the use of melarsomine dihydrochloride at this dose, frequency and route of administration in regions with a heavy, mixed *Trypanosoma* spp. burden cannot be recommended for treatment of equine infections. Melarsomine dihydrochloride is licensed in camels for treatment of *T*. *b*. *evansi* [30] where improved tissue penetration [66] may carry treatment benefits not assessed by this study.

Diminazene was non-inferior to isometamidium for treatment of *T*. *vivax* infections but not for *T*. *congolense* or *T*. *brucei* sp.. In equines the pathogenicity of *T*. *vivax* is speculated to be low and therefore this may be of minimal clinical significance [19]. In this population it was not possible to extract information to corroborate these observations due to the complex co-infection profile. On a whole animal basis (which accounts for co-infection status) there was no evidence from the PCR analysis that the use of isometamidium should be substituted for diminazene or melarsomine dihydrochloride.

The immediate reaction to isometamidium in donkeys was the most significant adverse reaction. A low therapeutic index and dose-dependent toxicity [67] related to an anticholinesterase effect [68] has been documented in other species, producing similar signs. The adverse reaction is associated with decreased serum cations (calcium, magnesium, sodium and potassium) [69] consistent with third space losses and occasional deaths have been reported in cattle [70]. The small stature of donkeys could render them more vulnerable to over-estimation of body weight. The dose selected for this study was the lowest used in equines [23–25]. Inter-specific differences in tolerance are also possible as drug metabolism varies between equines [35]. In this study although no animal died and the reactions were transient, the clinical signs were severe. At the current time, in the absence of alternative therapeutics, pre-treatment of donkeys with an parasympatholytic agent such as buscopan, atropine or glycopyrrolate may ameliorate adverse signs [71]. This is corroborated by experimental studies [72]. Division of the drug dose into two fractions given an hour apart, or reducing the total dose could both decrease the occurrence of adverse effects if they are related to peak plasma concentration. The use of intramuscular isometamidium administration was discounted in equines due to reported associated severe muscle necrosis [69]. Further work is required to investigate isometamidium pharmacokinetics in equines. The prevalence of adverse effects was otherwise low. Due to a high baseline prevalence of diarrhoea (17/639; 3%) and abortion (60/340; 18% of female equines) in this population, both of which are also reported clinical signs of trypanosomiasis, interpretation of other potential adverse responses to treatment requires caution. There have been no previous associations between the trypanocides used in this study and these clinical signs and their safety in pregnancy has been evaluated in other species with no evidence of increased risk (isometamidium [70], diminazene [73] and melarsomine dihydrochloride [61,74]). Previous studies have raised concerns regarding the use of diminazene in equines [26] especially donkeys where development of central nervous signs had been reported [75]. The current study suggests that diminazene can be used safely in equines at the lower dose of 3.5mg/kg i.m., but re-evaluation of higher doses may be useful in relation to *T*. *brucei* sp. treatment prior to onset of neurological signs.

New or recrudescing *Trypanosoma* spp. infections were more common in the isometamidium group than the diminazene group indicating shorter residual activity. Isometamidium is typically thought to have a long duration of activity even when given intra-venously due to prolonged release from secondary depots (mean elimination half-life is 135hrs in cattle) [76]. This has not been confirmed in equines but was supported here by the continued reduction in the number of post-treatment PCR positive animals from week 2 to week 3. New or recrudescing infections could indicate resistance [23] or differing pharmacokinetics in equines. Diminazene has a similar reported elimination half-life (74hrs [77] - 200 hrs [78]) to intravenous isometamidium in cattle and efficacy for up to 3 weeks [79]. The low new infection rate present in the diminazene group could support a sustained effect in equines. For diminazene, new infections were restricted to *T brucei* sp. consistent with the higher required therapeutic dose (7mg/kg) for this parasite [79]. These results challenge the use of a 'sanative pair' [80] approach in equines due to evidence of a shorter residual activity of isometamidium. Melarsomine dihydrochloride does not have any reported residual activity [61] and this is consistent with the highest rates of new infection observed in this group.

Limitations to the current investigation include the significant levels of variation present within the population when field cases are studied. Randomisation of treatment groups sought to minimise bias therefore starting parameters for the three treatment groups were similar. A placebo group would have been beneficial to account for varying parameters in waxing and waning chronic disease, but ethically this could not be justified. The high prevalence of piroplasmosis present in the region could also have complicated interpretation of post-treatment clinical signs as diminazene is known to have efficacy against piroplasms [81]. There are also limitations associated with utilising a non-quantitative PCR to assess infections post-treatment as changes in parasitaemia are only detected if they cross the threshold of detection. Positive results could also occur due to drug resistance (intrinsic/ acquired), inappropriate drug dose or dosing interval. The PCR assay used in this study does not differentiate between viable and non-viable *Trypanosoma* sp. DNA which is particularly pertinent following trypanocidal treatment. For parasites with significant tissue burdens (*T*. *vivax* and *T*. *brucei* sp.[82–84]) parasitaemia may be an insensitive marker of clinical effect leading to false negatives. Furthermore the presence of non-viable trypanosome DNA persisting in the sample following trypanocidal drug administration could give rise to a positive PCR reaction and lead to a false assessment of the level of drug resistance within the population.’

In conclusion, analysis of the treatment response on clinical evaluation, clinicopathological results and molecular diagnostics support the continued use of isometamidium as a first line trypanocide within this population with diminazene recommended as a second choice but question the use of a 'sanative pair' [80] approach in equines. Further investigation into the pharmacokinetics and duration of action of isometamidium when given intravenously to equines (horses, mules and donkeys) is necessary. The use of melarsomine dihydrochloride is not recommended in this region at this dose, frequency and route of administration. These observations highlight the importance of a multi-faceted approach to disease control as the rate of new infections is high. Currently the biggest limitation on equine trypanosomiasis treatment in this region of The Gambia is not drug efficacy but the absence of a coordinated approach. Before novel therapeutics are trialled it is essential that these problems are addressed; sporadic, non-targeted use in this environment will favour development of drug resistance. In the short term, coordination of large scale equine trypanocidal treatment contiguous with treatment of cattle may be beneficial.

## Supporting information

S1 Table Clinical parameters of treatment trial population (n = 162) subdivided by animal species. Clinical parameters of treatment trial population (n = 162) subdivided by animal species due to different reference ranges (horse or donkey). All individuals used for the purposes of the treatment trial were positive for at least one *Trypanosoma* sp. on PCR analysis. Values are presented as median and IQR. Signalment of the study population was representative of the sampled population and published census information of the general population [17,18]. *Denotes median value greater than published reference values. ^Denotes median value below published reference values.
(DOCX)

S2 Table Clinical parameters of the treatment population (n = 162) by treatment group. Clinical parameters of the treatment population (n = 162) (temperature, heart rate and respiration) given as median values with interquartile range) over the study period (week 1, 2 and 3) subdivided by trypanocidal drug group (melarsomine dihydrochloride (Cy), diminazene (Dim), isometamidium (Iso)). Data are presented by species (median and IQR) and categorised as within or out of reference range (proportion and percentage).
(DOCX)

S3 Table PCR status of the treatment trial population (n = 162). PCR status of the treatment trial population (n = 162) for *Trypanosoma sp (T*. *congolense*, *T*. *vivax*, *T*. *brucei sp*.*)* subdivided by trypanocidal drug (Cy = Melarsomine dihydrochloride, Dim = diminazene, Iso = isometamidium) and time point (week 1, week 2, week 3). New positives are defined as negative on week 1 but positive on week 2 by PCR. Results are presented as proportions (percentage).
(DOCX)

S4 Table Subjective assessment of the treatment trial population subdivided by trypanocidal drug and timepoint. Subjective assessment of the treatment trial population (n = 162) subdivided by trypanocidal drug (melarsamine dihydrochloride (Cy), diminazene (Dim), isometamidium (Iso)) and timepoint (week 1, week 2 or week 3). Results are presented as proportions (percentage).
(DOCX)

S5 Table Non-inferiority analysis comparing isometamidium to diminazene by *Trypanosoma* spp. Summarising the comparative success of the test drug (diminazene) in achieving negative PCR status for each tested *Trypanosoma* spp. when compared to the control (isometamidium). Results are presented as percentage (proportions), difference in percentage (95% CI) or risk ratio (95% CI).
(DOCX)

S6 Table Non-inferiority analysis comparing isometamidium to melarsomine dihydrochloride by *Trypanosoma* spp. Summarising the comparative success of the test drug (melarsomine dihydrochloride) in achieving negative PCR status for each tested *Trypanosoma* spp. when compared to the control (isometamidium). Results are presented as percentage (proportions), difference in percentage (95% CI) or risk ratio (95% CI).
(DOCX)

S7 Table Non inferiority analysis on whole animal PCR status. Summarising the comparative success of the test drugs (diminazene and melarsomine dihydrochloride) in achieving negative PCR status for all *Trypanosoma* spp. when compared to the control (isometamidium). Results are presented as percentage (proportions), difference in percentage (95% CI) or risk ratio (95% CI).
(DOCX)
