# Supplementary material for: Safety and efficacy of three trypanocides in confirmed field cases of trypanosomiasis in working equines in The Gambia: a prospective, randomised, non-inferiority trial
Source: PLoS Negl Trop Dis. 2019 Mar 22;13(3):e0007175. doi: 10.1371/journal.pntd.0007175 (PMC6447232; doi:10.1371/journal.pntd.0007175)
Supplement: S1 Table — Clinical parameters of treatment trial population (n = 162) subdivided by animal species due to different reference ranges (horse or donkey). All individuals used for the purposes of the treatment trial were positive for at least one Trypanosoma sp. on PCR analysis. Values are presented as median and IQR. Signalment of the study population was representative of the sampled population and published census information of the general population [17,18]. *Denotes median value greater than published reference values. ^Denotes median value below published reference values. (DOCX) [file pntd.0007175.s001.docx]

Table S1 Clinical parameters of treatment trial population (n=162) subdivided by animal species

| Parameter | | Donkey  n=107 | Horse  n=55 | Whole population  n=162 |
| --- | --- | --- | --- | --- |
| Presented for health check | | 32/107 (30%) | 4/55 (7%) | 36/162 (22%) |
| Estimated age (yrs) | | 7 (3-10) | 6 (2.5-12) | 7 (2.5-11) |
| Body condition score (0-5/5) | | 1.5 (1.5-2)^ | 1 (1-1.5)^ | 1.5 (1-2)^ |
| Estimated body weight (kg) | | 125 (115-146) | 228 (200-260) | 145 (120-200) |
| Gender | **Male** | 45/107 (42%) | 23/55 (42%) | 68/162 (42%) |
|  | **Female** | 62/107 (58%) | 32/55 (58%) | 94/162 (58%) |
| Temperature (°C) | | 38.2 (37.8-38.8)* | 37.8 (37.5-38.4) | 38.2 (37.7-38.8) |
| Pyrexia? | | 77/107 (72%) | 13/55 (24%) | 90/162 (56%) |
| Heart rate (bpm) | | 60 (56-68)* | 50 (43-60)* | 59 (50-64) |
| Tachycardia? | | 81/107 (76%) | 43/55 (78%) | 124/162 (77%) |
| Respiration (bpm) | | 32 (24-44)* | 32 (24-44)* | 32 (24-44)* |
| Haematocrit (%) | | 20 (16-23)^ | 20 (15-24)^ | 20 (16-23)^ |
| Anaemia? | | 103/107 (96%) | 54/55 (98%) | 157/162 (97%) |
| Total plasma protein (g/l) | | 82 (75-89)* | 82 (71-90)* | 82 (74-90)* |

Clinical parameters of treatment trial population (n=162) subdivided by animal species due to different reference ranges (horse or donkey). All individuals used for the purposes of the treatment trial were positive for at least one *Trypanosoma* sp. on PCR analysis.

Values are presented as median and IQR. Signalment of the study population was representative of the sampled population and published census information of the general population [18,19]

*Denotes median value greater than published reference values.

^Denotes median value below published reference values
