# Supplementary material for: Safety and efficacy of three trypanocides in confirmed field cases of trypanosomiasis in working equines in The Gambia: a prospective, randomised, non-inferiority trial
Source: PLoS Negl Trop Dis. 2019 Mar 22;13(3):e0007175. doi: 10.1371/journal.pntd.0007175 (PMC6447232; doi:10.1371/journal.pntd.0007175)
Supplement: S2 Table — Clinical parameters of the treatment population (n = 162) (temperature, heart rate and respiration) given as median values with interquartile range) over the study period (week 1, 2 and 3) subdivided by trypanocidal drug group (melarsomine dihydrochloride (Cy), diminazene (Dim), isometamidium (Iso)). Data are presented by species (median and IQR) and categorised as within or out of reference range (proportion and percentage). (DOCX) [file pntd.0007175.s002.docx]

Table S2 Clinical parameters of the treatment population (n=162) by treatment group

|  |  | Week 1 |  |  | Week 2 |  |  | Week 3 |  |  |
| --- | --- | --- | --- | --- | --- | --- | --- | --- | --- | --- |
| Parameter |  | **Cy n=58** | **Dim n=51** | **Iso n=53** | **Cy n=53** | **Dim n=48** | **Iso n=49** | **Cy n=51** | **Dim n=45** | **Iso n=46** |
| Temperature (°C) | **Horse** | 37.7  (37.4-39.2) | 37.9  (37.5-38.5) | 37.9  (37.4-38.2) | 38.4  (38.0-38.8) | 37.9  (37.7-38.2) | 37.8  (37.7-38.1) | 37.6  (37.4-38.3) | 37.7  (37.3-38.0) | 37.7  (37.3-38.0) |
|  | **>38.5°C** | 7/21  (33%) | 2/16  (13%) | 4/18  (22%) | 7/20  (35%) | 2/15  (13%) | 1/17  (6%) | 2/19  (11%) | 0/12  (0%) | 1/15  (7%) |
|  | **Donkey** | 38.3  (37.8-38.9) | 38.3  (37.7-38.8) | 38.2  (37.9-38.7) | 38.2  (38.0-38.6) | 38.3  (37.8-38.8) | 38.1  (37.7-38.6) | 37.8  (37.3-38.3) | 37.8  (37.5-38.3) | 37.8  (37.6-38.5) |
|  | **>37.8°C** | 26/37 (70%) | 24/35 (69%) | 27/36 (75%) | 28/34 (82%) | 23/33 (70%) | 20/33 (61%) | 15/32 (47%) | 15/32 (47%) | 14/29 (48%) |
| Heart rate | **Horse** | 50 (44-60) | 51 (40-60) | 47 (44-60) | 56 (47-60) | 54 (47-60) | 44 (40-52) | 52 (44-60) | 50 (48-56) | 48 (46-55) |
| (bpm) | **> 40bpm** | 17/21 (81%) | 11/16 (69%) | 15/18 (83%) | 17/20 (85%) | 12/14 (86%) | 9/17 (53%) | 18/19 (95%) | 12/12 (100%) | 12/15 (80%) |
|  | **Donkey** | 60 (56-68) | 60 (52-66) | 60 (56-68) | 62 (56-76) | 56 (52-64) | 60 (56-64) | 57 (52-64) | 56 (48-60) | 60 (56-63) |
|  | **> 53bpm** | 29/37 (78%) | 25/35 (71%) | 27/35 (77%) | 27/34 (79%) | 22/33 (66%) | 27/32 (84%) | 19/32 (59%) | 19/33 (57%) | 25/31 (81%) |
| Respiration | **Horse** | 30 (24-32) | 28 (24-40) | 30 (24-40) | 31 (25-39) | 32 (25-40) | 24 (20-32) | 28 (24-32) | 32 (27-43) | 28 (24-32) |
| (bpm) | **>30bpm** | 8/20  (40%) | 7/15  (47%) | 7/17  (42%) | 9/19  (47%) | 9/14  (64%) | 5/17  (29%) | 6/19  (32%) | 6/12  (50%) | 5/14  (36%) |
|  | **Donkey** | 40 (32-48) | 32 (24-40) | 32 (28-46) | 42 (32-50) | 36 (28-40) | 32 (28-40) | 36 (28-40) | 32 (28-40) | 32 (28-40) |
|  | **>30bpm** | 28/37 (76%) | 19/35 (54%) | 20/35 (57%) | 27/34 (79%) | 22/33 (66%) | 20/33 (61%) | 22/32 (69%) | 20/32 (63%) | 18/31 (58%) |

Clinical parameters of the treatment population (n=162) (temperature, heart rate and respiration) given as median values with interquartile range) over the study period (week 1, 2 and 3) subdivided by trypanocidal drug group (melarsomine dihydrochloride (Cy), diminazene (Dim), isometamidium (Iso)).

Data are presented by species (median and IQR) and categorised as within or out of reference range (proportion and percentage).
