# Supplementary material for: Safety and efficacy of three trypanocides in confirmed field cases of trypanosomiasis in working equines in The Gambia: a prospective, randomised, non-inferiority trial
Source: PLoS Negl Trop Dis. 2019 Mar 22;13(3):e0007175. doi: 10.1371/journal.pntd.0007175 (PMC6447232; doi:10.1371/journal.pntd.0007175)
Supplement: S3 Table — PCR status of the treatment trial population (n = 162) for Trypanosoma sp (T. congolense, T. vivax, T. brucei sp.) subdivided by trypanocidal drug (Cy = Melarsomine dihydrochloride, Dim = diminazene, Iso = isometamidium) and time point (week 1, week 2, week 3). New positives are defined as negative on week 1 but positive on week 2 by PCR. Results are presented as proportions (percentage). (DOCX) [file pntd.0007175.s003.docx]

**Table S3 PCR status of the treatment trial population (n=162)**

|  |  | Week 1 |  |  | Week 2 |  |  | Week 3 |  |  |
| --- | --- | --- | --- | --- | --- | --- | --- | --- | --- | --- |
| *Trypanosoma* sp. | **PCR** | **Cy**  ***n=58*** | **Dim**  ***n=51*** | **Iso**  ***n=53*** | **Cy**  ***n=53*** | **Dim**  ***n=48*** | **Iso**  ***n=49*** | **Cy**  ***n=51*** | **Dim**  ***n=45*** | **Iso**  ***n=46*** |
| *T. congolense* | **Positive** | 41/58  (71 %) | 35/51  (68 %) | 34/53  (64 %) | 27/53  (51 %) | 4/48  (8 %) | 7/49  (14 %) | 29/51  (57 %) | 6/45  (13 %) | 0/46  (0 %) |
|  | **New positive** |  |  |  | 2/53  (4 %) | 0/48  (0 %) | 0/49  (0 %) | 2/51  (4 %) | 0/45  (0%) | 1/46  (2 %) |
|  | **Negative** | 17/58  (29 %) | 16/51  (31 %) | 19/53  (36 %) | 24/53  (45 %) | 44/48  (92 %) | 42/49  (86 %) | 20/51  (40 %) | 39/45  (87 %) | 45/46  (98 %) |
| *T. vivax* | **Positive** | 26/58  (45 %) | 23/51  (45 %) | 31/53  (58 %) | 17/53  (32 %) | 2/48  (4 %) | 2/49  (4 %) | 14/51  (27 %) | 0/45  (0 %) | 2/46  (4 %) |
|  | **New positive** |  |  |  | 3/53  (6 %) | 0/48  (0 %) | 0/49  (0 %) | 3/51  (6 %) | 0/45  (0 %) | 0/46  (0 %) |
|  | **Negative** | 32/58  (55 %) | 28/51  (55 %) | 22/53  (42 %) | 33/53  (62 %) | 46/48  (96 %) | 47/49  (96 %) | 34/51  (67 %) | 45/45  (100 %) | 44/46  (96 %) |
| *T. brucei* sp. | **Positive** | 12/58  (21 %) | 11/51  (22 %) | 15/53  (28 %) | 2/53  (4 %) | 1/48  (2 %) | 2/49  (4 %) | 4/51  (8 %) | 1/45  (2 %) | 0/46  (0 %) |
|  | **New positive** |  |  |  | 1/53  (2 %) | 0/48  (0 %) | 1/49  (2 %) | 0/51  (0 %) | 0/45  (0%) | 2/46  (4 %) |
|  | **Negative** | 46/58  (79 %) | 40/51  (78 %) | 38/53  (72 %) | 50/53  (94 %) | 47/48  (98 %) | 46/49  (94 %) | 47/51  (92 %) | 44/45  (98 %) | 44/46  (96 %) |

PCR status of the treatment trial population (n=162) for *Trypanosoma sp (T. congolense, T. vivax, T. brucei sp.)* subdivided by trypanocidal drug (Cy= Melarsomine dihydrochloride, Dim= diminazene, Iso=isometamidium) and time point (week 1, week 2, week 3).

New positives are defined as negative on week 1 but positive on week 2 by PCR.

Results are presented as proportions (percentage).
