# Supplementary material for: Safety and efficacy of three trypanocides in confirmed field cases of trypanosomiasis in working equines in The Gambia: a prospective, randomised, non-inferiority trial
Source: PLoS Negl Trop Dis. 2019 Mar 22;13(3):e0007175. doi: 10.1371/journal.pntd.0007175 (PMC6447232; doi:10.1371/journal.pntd.0007175)
Supplement: S4 Table — Subjective assessment of the treatment trial population (n = 162) subdivided by trypanocidal drug (melarsamine dihydrochloride (Cy), diminazene (Dim), isometamidium (Iso)) and timepoint (week 1, week 2 or week 3). Results are presented as proportions (percentage). (DOCX) [file pntd.0007175.s004.docx]

Table S4 Subjective assessment of the treatment trial population subdivided by trypanocidal drug and timepoint.

|  |  | Week 1 |  |  | Week 2 |  |  | Week 3 |  |  |
| --- | --- | --- | --- | --- | --- | --- | --- | --- | --- | --- |
| Parameter | **Assessment** | **Cy n=58** | **Dim n=51** | **Iso n=53** | **Cy n=53** | **Dim n=48** | **Iso n=49** | **Cy n=51** | **Dim n=45** | **Iso n=46** |
| *Demeanour* | **BAR** | 12/58  (21 %) | 6/51  (12 %) | 14/53  (26 %) | 19/53  (36 %) | 17/48  (35 %) | 26/49  (53 %) | 20/51  (39 %) | 22/45  (49 %) | 26/46  (57 %) |
|  | **QAR** | 36/58  (62 %) | 26/51  (51 %) | 22/53  (42 %) | 32/53  (60 %) | 26/48  (54 %) | 22/49  (45 %) | 27/51  (53 %) | 20/45  (44 %) | 20/46  (43 %) |
|  | **Dull** | 6/58  (10 %) | 13/51  (25 %) | 15/53  (28 %) | 2/53  (4 %) | 2/48  (4 %) | 1/49  (2 %) | 4/51  (8 %) | 2/45  (4 %) | 0/46  (0 %) |
| *BCS* | **≧2** | 19/58  (32 %) | 15/51  (29 %) | 16/53  (30 %) | 20/53  (38 %) | 18/48  (38 %) | 20/49  (41 %) | 23/51  (45 %) | 23/45  (51 %) | 26/46  (57 %) |
| *0-5/5* | **1.5** | 25/58  (43 %) | 22/51  (43 %) | 22/53  (42 %) | 32/53  (60 %) | 24/48  (50 %) | 24/49  (49 %) | 22/51  (43 %) | 20/45  (44 %) | 19/46  (41 %) |
|  | **1** | 10/58  (17 %) | 10/51  (20 %) | 10/53  (19 %) | 2/53  (4 %) | 4/48  (8 %) | 4/49  (8 %) | 4/51  (8 %) | 2/45  (4 %) | 3/46  (7 %) |
|  | **0.5** | 3/58  (5 %) | 4/51  (8 %) | 4/53  (8 %) | 0/53  (0 %) | 1/48  (2 %) | 2/49  (4 %) | 0/51  (0 %) | 0/45  (0 %) | 3/46  (7 %) |

Subjective assessment of the treatment trial population (n=162) subdivided by trypanocidal drug (melarsamine dihydrochloride (Cy), diminazene (Dim), isometamidium (Iso)) and timepoint (week 1, week 2 or week 3). Results are presented as proportions (percentage).
