# Supplementary material for: Safety and efficacy of three trypanocides in confirmed field cases of trypanosomiasis in working equines in The Gambia: a prospective, randomised, non-inferiority trial
Source: PLoS Negl Trop Dis. 2019 Mar 22;13(3):e0007175. doi: 10.1371/journal.pntd.0007175 (PMC6447232; doi:10.1371/journal.pntd.0007175)
Supplement: S5 Table — Summarising the comparative success of the test drug (diminazene) in achieving negative PCR status for each tested Trypanosoma spp. when compared to the control (isometamidium). Results are presented as percentage (proportions), difference in percentage (95% CI) or risk ratio (95% CI). (DOCX) [file pntd.0007175.s005.docx]

## Table S5 Non-inferiority analysis comparing isometamidium to diminazene by *Trypanosoma* spp.

| Measure of success | Time point | Test drug (diminazene)  % Success (negative PCR/ no. of equines) | Control drug (Isometamidium)  % Success (negative PCR/no. of equines) | Difference in % success (Control-test) with 95% CI | Risk ratio (test/control) with 95% CI |
| --- | --- | --- | --- | --- | --- |
| Negative *T. congolense* PCR | Week 2 | 87.5 % (28/32) | 78.0 % (25/32) | -9.5 (-28, 9) | 1.11 (0.89-1.39) |
|  | Week 3 | 81.0 % (25/31) | 100.0 % (31/31) | 19 (5, 33) | 0.81 (0.68-0.96) |
| Negative *T. vivax* PCR | Week 2 | 91.0 % (20/22) | 93.0 % (27/29) | 2 (-13, 17) | 0.97 (0.83-1.15) |
|  | Week 3 | 100 % (21/21) | 92.5 % (25/27) | -7.5 (-17, 2) | 1.08 (0.97-1.20) |
| Negative *T. brucei sp.* PCR | Week 2 | 91 % (10/11) | 86.5 % (13/15) | -4.5 (-28, 2) | 1.04 (0.79-1.36) |
|  | Week 3 | 91 % (10/11) | 100 % (12/12) | 9 (-8, 26) | 0.91 (0.75-1.10) |

Summarising the comparative success of the test drug (diminazene) in achieving negative PCR status for each tested *Trypanosoma* spp. when compared to the control (isometamidium)*.* Results are presented as percentage (proportions), difference in percentage (95% CI) or risk ratio (95% CI).
