# Supplementary material for: Safety and efficacy of three trypanocides in confirmed field cases of trypanosomiasis in working equines in The Gambia: a prospective, randomised, non-inferiority trial
Source: PLoS Negl Trop Dis. 2019 Mar 22;13(3):e0007175. doi: 10.1371/journal.pntd.0007175 (PMC6447232; doi:10.1371/journal.pntd.0007175)
Supplement: S7 Table — Summarising the comparative success of the test drugs (diminazene and melarsomine dihydrochloride) in achieving negative PCR status for all Trypanosoma spp. when compared to the control (isometamidium). Results are presented as percentage (proportions), difference in percentage (95% CI) or risk ratio (95% CI). (DOCX) [file pntd.0007175.s007.docx]

Table S7 Non inferiority analysis on whole animal PCR status

| Test drug | Time point | % Success  (negative PCR/  no. of equines) | Control drug (Isometamidium)  % Success (negative PCR/no. equines) | Difference in % success (Control-test)  with 95 % CI | Risk ratio (test/control) with 95 % CI |
| --- | --- | --- | --- | --- | --- |
| Diminazene | Week 2 | 87.5 % (42/48) | 79.6 % (39/49) | -7.9 (-23, 7) | 1.10 (0.92-1.31) |
|  | Week 3 | 84.4 % (38/45) | 93.5 % (43/46) | 9 (-4, 21) | 0.90 (0.78-1.04) |
| Melarsomine dihydrochloride | Week 2 | 22.6 % (12/53) | 79.6 % (39/49) | 57 (41, 73) | 0.28 (0.17-0.48) |
|  | Week 3 | 17.6 % (9/51) | 93.5 % (43/46) | 73.9 (61, 87) | 0.21 (0.12-0.37) |

Summarising the comparative success of the test drugs (diminazene and melarsomine dihydrochloride) in achieving negative PCR status for all *Trypanosoma* spp. when compared to the control (isometamidium)*.* Results are presented as percentage (proportions), difference in percentage (95% CI) or risk ratio (95% CI).
